# Novel *N*-Substituted 2-(2-(Adamantan-1-yl)-1*H*-Indol-3-yl)-2-Oxoacetamide Derivatives: Synthesis and Biological Evaluation

**DOI:** 10.3390/molecules21050530

**Published:** 2016-05-05

**Authors:** Hong-Yu Hu, Xu-Dong Yu, Fei Wang, Chun-Rong Lin, Jin-Zhang Zeng, Ying-Kun Qiu, Mei-Juan Fang, Zhen Wu

**Affiliations:** School of Pharmaceutical Sciences and the Key Laboratory for Chemical Biology of Fujian Province, Xiamen University, South Xiang-An Road, Xiamen 361102, China; huhongyu22@126.com (H.-Y.H.); yuxudong91@163.com (X.-D.Y.); tjdxwangfei@163.com (F.W.); reyiai@163.com (C.-R.L.); jzzeng@xmu.edu.cn (J.-Z.Z.); qyk@xmu.edu.cn (Y.-K.Q.)

**Keywords:** 2-(2-(adamantan-1-yl)-1*H*-indol-3-yl)-2-oxoacetamidederivatives, poly ADP-ribose polymerase (PARP), caspase-8, apoptosis

## Abstract

In this study, a series of novel *N*-substituted 2-(2-(adamantan-1-yl)-1*H*-indol-3-yl)-2-oxoacetamide derivatives were synthesized, and evaluated for their cytotoxicity in human cell lines including Hela (cervical cancer), MCF7 (breast cancer ) and HepG2 (liver cancer). Several compounds were found to have potent anti-proliferative activity against those human cancer cell lines and compound **5r** showed the most potent biological activity against HepG2 cells with an IC_50_ value of 10.56 ± 1.14 μΜ. In addition, bioassays showed that compound **5r** induced time-dependent and dose-dependent cleavage of poly ADP-ribose polymerase (PARP), and also induced a dose-dependent increase in caspase-3 and caspase-8 activity, but had little effect on caspase-9 protease activity in HepG2 cells. These results provide evidence that **5r**-induced apoptosis in HepG2 cell is caspase-8-dependent.

## 1. Introduction

Despite continued research efforts, cancer remains one of the biggest threats to human health, and it was estimated to be responsible for 15% of all global deaths in 2010 [1]. Current treatment for tumors generally involves surgical resection (if possible), followed by radiotherapy and chemotherapy, with the most common chemotherapies, according to Cancer Research UK, being temozolomide, procarbazine, carmustine, lomustine and vincristine [2,3,4,5]. However these drugs alone simply aren’t sufficient for long-term treatment because of the rapid chemoresistance developed by many cancers [6]. Multidrug resistance (MDR) is a major obstacle to successful cancer treatment [7,8]. This has driven the development of a variety of new anticancer agents with more potent, high specific and low cytotoxic properties [9].

The indole moiety has been described as a privileged structure as it appears extensively in many unrelated areas of biology and medicine, and depending on the substituents, can have a diverse range of effects [10]. The indole ring system as one of the most ubiquitous heterocycles in Nature, and has been becoming an important structural component in many pharmaceutical agents, such as antidepressant [11], anticonvulsant [12], antifungal [13], antiviral [14] and anti-inflammatory [15], and particularly new antitumor agents [16,17,18,19]. Indole-3-glyoxylamide compounds, as a new class of indole derivatives which have anti-tumor [20,21,22], anti-viral [23], anti-bacterial [24,25], anti-HIV [26], adenosine regulating receptor function [27] and other biological activities, have become one of the most important chemical entities in the field of pharmaceutical research. Thus, we synthesized a novel class of *N*-substituted 2-(2-(adamantan-1-yl)-1*H*-indol-3-yl)-2-oxoacetamide derivatives and evaluated their *in vitro* anti-proliferative activity against human breast (MCF7), cervical (Hela) and liver (HepG2) cancer cells. To explain the molecular mechanisms mediating the induction of apoptosis by this class of compounds, the activation of caspase-3, caspase-8 and caspase-9 was further examined using a caspase activity assay kit in HepG2 cells after treatment with compound **5r**.

## 2. Results and Discussion

### 2.1. Chemistry

**Scheme 1:**
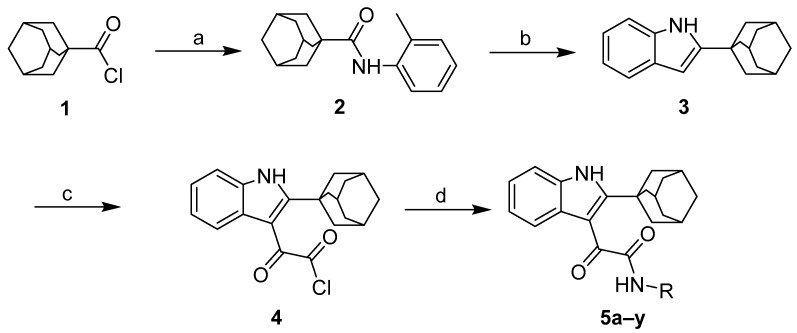
Synthesis of *N*-substituted 2-(2-(adamantan-1-yl)-1*H*-indol-3-yl)-2-oxoacetamide. *Reagents and conditions*: (a) toluene, *o*-toluidine, K_2_CO_3_, r.t., 4 h; (b) *n*-BuLi, THF, 0 °C, N_2_, 3 h; (c) ether, oxalyl chloride, 0 °C, 2 h; (d) toluene, *o*-toluidine, K_2_CO_3_, r.t., 4 h.

The preparation of the *N*-substituted 2-(2-(adamantan-1-yl)-1*H*-indol-3-yl)-2-oxoacetamide derivatives **5a**–**y** is outlined in Scheme 1. First, we synthesized 2-adamantane-1*H*-indol-5-amine (**3**). The general chemistry used in the synthesis of **3** was adapted from reported previously methods [28,29]. Briefly, treatment of adamantane-1-carbonyl chloride (**1**) with *o*-Toluidine resulted in *N*-*o*-tolylcycloadamantanecarboxamide (**2**), followed by dropwise addition of 1.4 or 1.6 M *n*-BuLi in hexane to get the compound **3**. Then the intermediate 2-(2-(adamantan-1-yl)-1*H*-indol-3-yl)-2-oxoacetyl chloride (**4**) was easily formed by the reaction of **3** and oxalyl chloride in anhydrous diethyl ether. Finally, the reaction of compound **4** with various substituted amines in toluene at 60 °C for 4 h afforded the title compounds **5a**–**y**. The structures of all the target compounds were confirmed by their spectral data (HRMS, ^1^H-NMR and ^13^C-NMR).

### 2.2. Biological Evaluation

#### 2.2.1. Cytotoxicity in Human Tumor Cell Lines

All synthesized compounds were evaluated for their *in vitro* anti-proliferative activity against three human cancer cell lines: MCF-7 (breast cancer cells), Hela (cervical cancer cells), HepG2 (liver cancer cells). The concentration required for 50% inhibition of cell viability (IC_50_) was calculated and the results are given in Table 1. Most compounds showed a moderate anti-proliferative activity against all tested cell lines, whereas compounds **5a**, **5b**, **5e** and 5**j** exhibited relatively weak activity against one or all tested cell lines, with IC_50_ values over 100 μM. On the other hand, **5i**, **5p**, **5r** and **5y** exhibited good cytotoxicity against HepG2, whereas compound **5f**, with an IC_50_ value of 17.65 ± 1.54 μM, was more active against Hela.

Interestingly, compound **5a** with a *N*-cyclopropyl group exhibited the lowest cytotoxic activity against these three cancer lines, with IC_50_ values of over 100 μM, however introduction of single ring aryl group at the *N*-position such as in compounds **5c**, **5d** and **5f** caused an increase in cytotoxic activity, and among them compound **5f** with a *N*-benzene substituent had the strongest cytotoxic activity against the Hela cell line, with an IC_50_ value of 17.65 ± 1.54 μM. These data suggested the presence of alkyl ring at the *N*′-position had an important relationship with anti-proliferative activity. To investigate the impact of the substituents on the *N*-phenyl ring, halogen (F, Cl, Br or I), methyl (CH_3_), trifluoromethyl (CF_3_), methoxy (OCH_3_), cyano (CN), nitro (NO_2_) and/or methoxy formyl (COOCH_3_) was introduced (compounds **5g**–**u**). Substitution at the *N*-phenyl ring was unhelpful, except for compound **5r** with *m*-Cl and *p*-F on the *N*-phenyl ring as shown in Table 1. In addition, replacement of phenyl with pyridyl (**5v** and **5w**), pyrimidine (**5x**) or isoxazole (**5y**) resulted in similar cellular anti-proliferative activity and the introduction of a fused aromatic ring group (compound **5e**) or alkyl group (**5b**) between nitrogen atom and phenyl group was generally unfavorable for anti-proliferative properties.

Based on data collected from three independent experiments, compound **5r** showed the most cytotoxic activity against Hela, MCF-7 and HepG2 cell lines with IC_50_ values of 16.12 ± 1.54, 12.54 ± 1.15 and 10.56 ± 1.14 μM, respectively, so we used compound **5r** for further biological activity studies.

#### 2.2.2. Growth Inhibitory Activity of **5r** in HepG2

**Figure 1:**
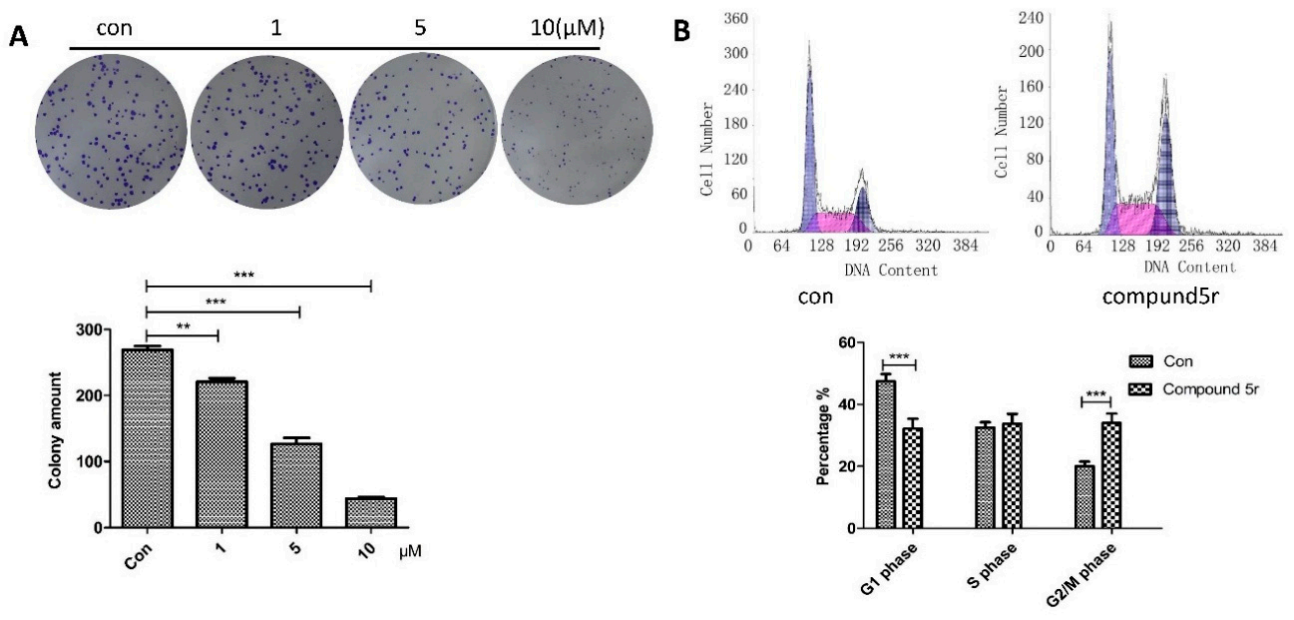
Effects of compound **5r** on colony-formation and cell-cycle distribution in HepG2 cells. (**A**) Compound **5r** inhibits the colony-forming activity of HepG2 cells. Cells were grown in 6-well plates for 6 days and treated with compound **5r** (1, 5 and 10 μM). Colonies of HepG2 cells decreased after compound **5r** treatment in a dose dependent manner (** *p* < 0.01 and *** *p* < 0.001 compared with the control, *t-*test); (**B**) A flow cytometry assay was performed to examine cell cycle arrest. HepG2 cells were treated with 10 μM compound **5r** for 12 h. Compound **5r** reduced a significant accumulation of cell-cycle arrest (*** *p* < 0.001 compared with the control, *t-*test).

As cell proliferation depends on cell division which is regulated by the cell cycle, absence of normal cell-cycle control is a hallmark of cancer [30]. Cell cycle-related proteins have been as the therapeutic targets against cancer and lots of small molecules were developed as potent antitumor agents, such as microtubule-targeting agents, and cyclin-dependent kinases, aurora kinases and polo-like kinases inhibitors [31,32,33,34]. The present study sought to determine how compound **5r** inhibited HepG2 cell growth. Microscopic analysis indicated that the colonies of HepG2 cells decreased after compound **5r** treatment in a dose dependent manner, compared to the control group (Figure 1A). Furthermore, flow cytometry was performed to examine cell cycle inhibition after 12 h of compound **5r** treatment. The results revealed a significant accumulation of cell-cycle arrest, with a decrease in G0/G1 phase and an increase in G2/M phase arrest at 12 h, indicating that inhibitory activity of compound **5r** was associated with disruption of cell cycle (Figure 1B). Meanwhile, the effects of compound **5r** on colony-formation and cell-cycle distribution in Hela and MCF-7 cells were also examined (see Supplementary Materials).

#### 2.2.3. Inducing Apoptosis in HepG2 Cells

**Figure 2:**
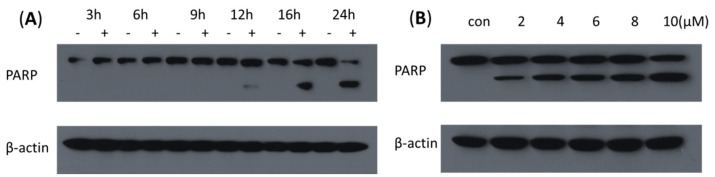
Apoptosis induction by compound **5r** in HepG2. Western blot analysis of PARP in HepG2 cells treated with 10 μM compound **5r** for different periods of time (0, 3, 6, 9, 12, 16 and 24 h) (**A**); and different concentration of compound **5r** (2, 4, 6, 8 and 10 μM) for 24 h (**B**).

Apoptosis, as a fundamental biological process, plays an important role in cell growth, development and tissue homeostasis [35,36,37]. Deregulation in apoptotic cell contributes to many diseases, including cancer, neurodegenerative disorders and cardiovascular diseases [38,39]. Apoptosis can be further characterized as cell death accompanied by the activation of a unique family of cysteine-dependent specific proteases called caspases [40]. Two major signaling pathways induce apoptotic cell death: the mitochondrial pathway and the death receptor pathway [41]. The former relies on mitochondrial depolarization and permeability increase in response to a variety of cellular stresses, including DNA damage, growth factor deprivation, ER stress, thus resulting in the release of cytochrome C, then initiating formation of an APAF-1/caspase-9 complex and activation of downstream executionary caspases, including caspase-3, caspase-6, and caspase-7, and finally leading to cell death [42,43]. The second pathway is activated predominantly by the binding of death receptor ligands, including tumor necrosis factor-α (TNF-α), fas ligand (CD95) and tumor necrosis factor-related apoptosis inducing ligand (TRAIL) to their respective death receptors, then initiating the assembly of large macromolecular complexes that recruit and activate caspase-8, which further cleave and activate caspase-3 for apoptosis [44]. In our work, it was demonstrated that compound **5r** could induce poly ADP-ribose polymerase (PARP) cleavage, which served as a marker of cells undergoing apoptosis, in a time- and dose-dependent manner (Figure 2A,B).

**Figure 3:**
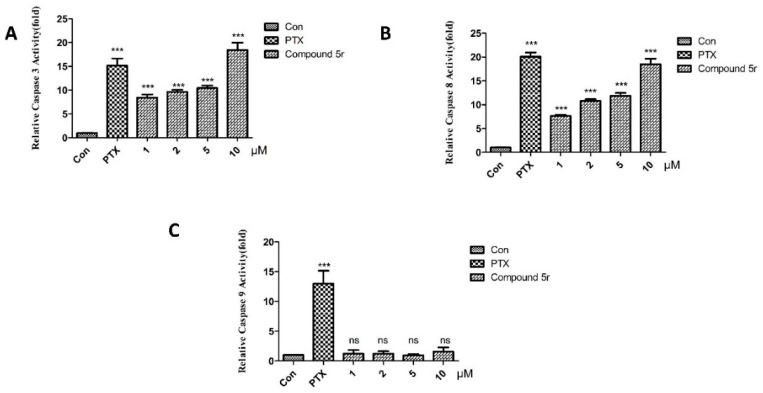
Induction of caspase-3, caspase-8 and caspase-9 by compound **5r** in HepG2. Cells were treated with different concentration of compound **5r** (1, 2, 5 and 10 μM) and analyzed by caspase-3 (**A**); caspase-8 (**B**) and caspase-9 (**C**) activity assay kit. As a positive control, HepG2 cells were treated with (PTX, 1 μM) and analyze. Compound **5r** significantly stimulated caspase-3 and caspase-8 protease activities in HepG2 cells, yet had little effect on caspase-9 protease activity (*** *p* < 0.001 and ns *p* > 0.05 compared with the control, *t*-test).

The PARP is one of the important targets of caspase-3, which is also downstream of capase-8 and caspase-9 [45]. Therefore, the activation of caspase-3, caspase-8 and caspase-9 was examined in HepG2 cells using a caspase activity assay kit after treatment with compound **5r**. It was shown that compound **5r** significantly stimulated caspase-3 and caspase-8 protease activities in HepG2 cells, yet had little effect on caspase-9 protease activity (Figure 3). The results suggested that compound **5r** resulted in the caspases-3 activation and PARP cleavage by activating caspase-8, finally leading to cell death.

## 3. Materials and Methods 

### 3.1. General Information

All reagents were commercially available and used without further purification unless otherwise indicated. Reaction mixtures were magnetically stirred and monitored by thin-layer chromatography (TLC) on Yantai Wish chemical products Co., Ltd. (Yantai, China) silica gel 60F-254 by fluorescence quenching under UV light. All of the final compounds were purified by column chromatography. ^1^H-NMR and ^13^C-NMR spectra were recorded on an AV2 600 MHz spectrometer (Bruker Biospin, Swiss). Chemical shifts (δ) were given in parts per million (ppm) relative to tetramethylsilane (TMS) as an internal standard. Multiplicities were abbreviated as follows: single (s), doublet (d), doublet-doublet (dd), doublet-triplet (dt), triplet (t), triplet-triplet (tt), triplet-doublet (td), quartet (q), quartet-doublet (qd), multiplet (m), and broad signal (br s). Positive mode high-resolution mass spectral (HRMS) data were acquired using electrospray ionization (ESI) on a Q Exactive LC-MS/MS instrument (Thermo Fisher Scientific Inc., Waltham, MA, USA) with UV detection at 254 nm.

### 3.2. Synthesis of N-o-Tolylcycloadamantanecarboxamide *(**2**)*

A mixture of *o*-toluidine (10.0 mmol), adamantane-1-carbonyl chloride (10.0 mmol) and anhydrous potassium carbonate (7 mmol) in toluene (50 mL) was stirred at room temperature for 4 h. The resulting solids was filtered off and stirred at room temperature for 1.5 h with H_2_O (50 mL). After completion of the reaction, the solid was filtered off and recrystallized from the appropriate solvent. White solid product, yield 75.5%. ^1^H-NMR (CDCl_3_): δ 7.88 (d, *J* = 8.07 Hz, 1H), 7.18–7.24 (m, 2H), 7.17 (d, *J* = 7.52 Hz, 1H), 7.05 (dt, *J* = 1.10, 7.43 Hz, 1H), 2.26 (s, 3H), 2.11 (br s, 3H), 1.99 (d, *J* = 2.57 Hz, 6H), 1.77 (q, *J* = 12.29 Hz, 6H). ESI-HRMS (+): *m*/*z* [M + H]^+^ calculated for C_18_H_23_NO^+^, 270.1852, found 270.1853; [M + Na]^+^ calculated C_18_H_23_NONa^+^, 292.1672, found 292.1669.

### 3.3. Synthesis of 2-Adamantane-1H-indole *(**3**)*

A stirred solution of compound 2 (10 mmol) in 50 mL of tetrahydrofuran (THF) under a N_2_ atmosphere was maintained at an internal temperature of –5 to 5 °C and treated dropwise with 0.1–0.15 mol of 2.5 M *n*-BuLi in hexane. The mixture was stirred at ambient temperature for 3 h, cooled in an ice bath, and treated dropwise with 2 M HCl (12 mL). Then, the organic layer was separated and the aqueous layer washed with C_6_H_6_. The combined organic layer was dried with anhydrous MgSO_4_, filtered, and concentrated *in vacuo*. The residue was recrystallized from the appropriate solvent. White solid product, yield 56.5%. ^1^H-NMR (CDCl_3_): δ 7.30 (d, *J* = 7.89 Hz, 1H), 7.18 (dd, *J* = 0.55, 8.07 Hz, 1H), 6.88 (dt, *J* = 1.10, 7.52 Hz, 1H), 6.77–6.83 (m, 1H), 5.98 (d, *J* = 0.55 Hz, 1H), 1.97 (d, *J* = 2.57 Hz, 3H), 1.93 (d, *J* = 2.93 Hz, 6H), 1.73 (br s, 6H). ESI-HRMS (+): *m*/*z* [M + H]^+^ calculated for C_18_H_21_N^+^, 252.1747, found 252.1748; [M + Na]^+^ calculated for C_18_H_21_NNa^+^, 274.1566, found 274.1568.

### 3.4 General Procedure for Synthesis of ***5a**–**y** [23,46]*

A dry 50 mL capacity carousel reaction tube was charged with compound **3** (1.5 mmol), and this starting material was dissolved in dry ether (12 mL). Oxalyl chloride (1.65 mmol) was added and the mixture stirred at room temperature for 2 h, the solid was filtered off. Then compound **4** was used, without purification, for the next reaction. To a solution of **4** (1 mmol) and a substituted amine (1 mmol) in toluene (10 mL) was added K_2_CO_3_ (1.0 mmol), and the mixture stirred at room temp. After completion of the reaction, the mixture was concentrated and purified by column chromatography using appropriate mixtures of CH_2_Cl_2_/MeOH to give compounds **5a**–**y**.

*N-Cyclopropyl-2-(2-adamantane-3H-indol-3-yl)-2-oxoacetamide* (**5a**): yellow solid, yield 87.0%. ^1^H-NMR (DMSO-*d*_6_): δ 8.69 (d, *J* = 4.58 Hz, 1H), 7.53 (d, *J* = 7.52 Hz, 1H), 7.48 (d, *J* = 7.52 Hz, 1H), 7.24–7.27 (m, 1H), 2.89 (qt, *J* = 3.97, 7.61 Hz, 1H), 2.21 (br s, 6H), 2.08 (br s, 3H), 1.69–1.88 (m, 6H), 0.71–0.79 (m, 2H), 0.52–0.61 (m, 2H). ^13^C-NMR (DMSO-*d*_6_): δ 186.9, 170.3, 157.8, 134.7, 129.4, 122.3, 121.9, 119.8, 112.8, 108.2, 38.8, 36.5, 36.3, 28.3, 22.7, 21.5, 5.8.ESI-HRMS (+): *m*/*z* [M + H]^+^ calculated for C_23_H_26_N_2_O_2_^+^, 363.2067, found 363.2065; [M + Na]^+^ calculated for C_23_H_26_N_2_O_2_Na^+^, 385.1886 found 385.1888.

*N-(4-Fluorobenzyl)-2-(2-adamantane-1H-indol-3-yl)-2-oxoacetamide* (**5b**): yellow solid, yield 87.5%. ^1^H-NMR (DMSO-*d*_6_): δ 9.17 (t, *J* = 6.05 Hz, 1H), 7.51 (d, *J* = 8.07 Hz, 1H), 7.42 (dd, *J* = 5.69, 8.44 Hz, 2H), 7.30 (d, *J* = 8.07 Hz, 1H), 7.18–7.23 (m, 2H), 7.09–7.13 (m, 1H), 6.92–6.96 (m, 1H), 4.46 (d, *J* = 6.05 Hz, 2H), 2.22 (br s, 6H), 2.07 (br s, 3H), 1.73–1.84 (m, 6H). ^13^C-NMR (DMSO-*d*_6_): δ 186.5, 169.2, 161.8 (d, *J* = 243.2 Hz), 158.4, 135.4 (d, *J* = 3.3 Hz), 130.3 (d, *J* = 8.8 Hz), 129.4, 128.4, 125.8, 121.9 (d, *J* = 63.8 Hz), 119.8, 115.6, 115.4, 112.9, 108.0, 41.6, 38.8, 36.6, 36.4, 28.4. ESI-HRMS (+): *m*/*z* [M + H]^+^ calculated for C_27_H_27_FN_2_O_2_^+^, 431.2129, found 431.2126; [M + Na]^+^ calculated for C_27_H_27_FN_2_O_2_ Na^+^, 453.1949, found 453.5194.

*2-(2-Adamantane-1H-indol-3-yl)-2-oxo-N-(2-(pyridin-3-yl)ethyl)acetamide* (**5c**): yellow solid, yield 86.0%. ^1^H-NMR (DMSO-*d*_6_): δ 8.75 (t, *J* = 5.41 Hz, 1H), 8.53 (d, *J* = 4.22 Hz, 1H), 7.71 (dt, *J* = 1.47, 7.61 Hz, 1H), 7.51 (d, *J* = 7.89 Hz, 1H), 7.34 (t, *J* = 8.16 Hz, 2H), 7.24 (dd, *J* = 5.23, 6.88 Hz, 1H), 7.12 (t, *J* = 7.43 Hz, 1H), 7.04 (t, *J* = 7.52 Hz, 1H), 3.67 (q, *J* = 6.97 Hz, 2H), 3.00–3.06 (m, 2H), 2.20 (br s, 6H), 2.07 (br s, 3H), 1.72–1.84 (m, 6H). ^13^C-NMR (DMSO-*d*_6_): δ 187.1, 169.0, 158.4, 149.6, 136.9, 134.6, 127.9, 123.7, 122.2, 122.1, 121.9, 119.9, 112.7, 108.2, 40.0, 38.9, 38.7, 37.3, 36.5, 36.3, 28.4. ESI-HRMS (+): *m*/*z* [M + H]^+^ calculated for C_27_H_29_N_3_O_2_^+^, 428.2333, found 428.2330; [M + Na]^+^ calculated for C_27_H_29_N_3_O_2_Na^+^, 450.2152, found 450.2150.

*2-(2-Adamantane-1H-indol-3-yl)-2-oxo-N-(2-(thiophen-2-yl)ethyl)acetamide* (**5d**): yellow solid, yield 88.9%. ^1^H-NMR (DMSO-*d*_6_): δ 11.69 (br s, 1H), 8.82 (t, *J* = 5.50 Hz, 1H), 7.51 (d, *J* = 8.07 Hz, 1H), 7.34–7.40 (m, 2H), 7.13 (t, *J* = 7.52 Hz, 1H), 7.04–7.09 (m, 1H), 6.97 (d, *J* = 3.30 Hz, 2H), 3.55 (q, *J* = 6.85 Hz, 2H), 3.05–3.13 (m, 2H), 2.21 (br s, 6H), 2.08 (br s, 3H), 1.73–1.83 (m, 6H). ^13^C-NMR (DMSO-*d*_6_): δ 167.1, 149.1, 137.8, 121.9, 114.7, 108.0, 107.6, 106.0, 104.7, 102.2, 100.1, 92.8, 88.4, 20.2, 19.0, 16.7, 16.5, 9.5, 8.5. ESI-HRMS (+): *m*/*z* [M + H]^+^ calculated for C_26_H_28_N_2_O_2_S^+^, 433.1944, found 433.1942; [M + Na]^+^ calculated for C_26_H_28_N_2_O_2_SNa^+^, 455.1764, found 455.1761.

*N-(2-(1H-Indol-2-yl)ethyl)-2-(2-adamantane-1H-indol-3-yl)-2-oxoacetamide* (**5e**): yellow solid, yield 87.9%. ^1^H-NMR (DMSO-*d*_6_): δ 10.89 (brs, 1H), 8.79 (br s, 1H), 7.50–7.61 (m, 2H), 7.33–7.44 (m, 2H), 7.22–7.25 (m, 1H), 7.16–7.19 (m, 1H), 7.09 (dd, *J* = 7.89, 16.14 Hz, 2H), 6.97–7.01 (m, 2H), 3.59 (d, *J* = 6.24 Hz, 2H), 2.99 (t, *J* = 7.15 Hz, 2H), 2.22 (br s, 6H), 2.08 (br s, 3H), 1.70–1.85 (m, 6H). ^13^C-NMR (DMSO-*d*_6_): δ 187.3, 169.0, 157.6, 136.8, 129.4, 128.7, 127.9, 127.6, 125.8, 123.3, 122.2, 121.8, 121.4, 119.9, 118.7, 118.7, 112.7, 111.9, 41.6, 38.9, 36.6, 36.3, 28.4, 25.3. ESI-HRMS (+): *m*/*z* [M + H]^+^ calculated for C_30_H_31_N_3_O_2_^+^, 466.2489, found 466.2487; [M + Na]^+^ calculated for C_30_H_31_N_3_O_2_Na^+^, 488.2308, found 488.2306.

*2-(2-Adamantane -3H-indol-3-yl)-2-oxo-N-phenylacetamide* (**5f**): yellow solid, yield 91.9% .^1^H-NMR (DMSO-*d*_6_): δ 7.72-7.82 (m, 2H), 7.54 (d, *J* = 8.07 Hz, 1H), 7.35–7.44 (m, 3H), 7.15 (t, *J* = 7.43 Hz, 1H), 7.10 (t, *J* = 7.52 Hz, 1H), 6.97-7.03 (m, 2H), 6.56 (dd, *J* = 1.10, 8.44 Hz, 1H), 6.44–6.52 (m, 1H), 2.28 (br s, 6H), 2.10 (br s, 3H), 1.74-1.88 (m, 6H). ^13^C-NMR (DMSO-*d*_6_): δ 184.7, 167.9, 149.1, 139.1, 129.5, 129.2, 124.4, 122.0, 121.9, 120.2, 119.2, 116.1, 114.3, 107.7, 38.8, 36.6, 36.6, 28.4. ESI-HRMS (+): *m*/*z* [M + H]^+^ calculated for C_26_H_26_N_2_O_2_^+^, 399.2067, found 399.2063; [M + Na]^+^ calculated for C_26_H_26_N_2_O_2_Na^+^, 421.1886, found 421.1882.

*2-(2-Adamantane-3H-indol-3-yl)-2-oxo-N-p-tolylacetamide* (**5g**): yellow solid, yield 86.5%. ^1^H-NMR (DMSO-*d*_6_): δ 10.68 (s, 1H), 7.66 (d, *J* = 8.25 Hz, 2H), 7.57 (d, *J* = 8.07 Hz, 1H), 7.41 (d, *J* = 8.07 Hz, 1 H), 7.21 (d, *J* = 8.25 Hz, 2H), 7.14 (t, *J* = 7.43 Hz, 1H), 7.02–7.06 (m, 1H), 2.30 (s, 3H), 2.27 (br s, 6H), 2.11 (br s, 3H), 1.73–1.87 (m, 6H). ^13^C-NMR (DMSO-*d*_6_): δ 185.6, 167.4, 158.5, 136.4, 134.7, 133.6, 129.9, 129.4, 122.4, 122.2, 120.2, 119.4, 113.0, 107.9, 38.7, 36.5, 36.4, 28.4, 21.0. ESI-HRMS (+): *m*/*z* [M + H]^+^ calculated for C_27_H_28_N_2_O_2_^+^, 413.2224, found 413.2226; [M + Na]^+^ calculated for C_27_H_28_N_2_O_2_Na^+^, 435.2043, found 435.2045.

*N-(2-Methoxyphenyl)-2-(2-adamantane-3H-indol-3-yl)-2-oxoacetamide* (**5h**): yellow solid, yield 91.5%. ^1^H-NMR (DMSO-*d*_6_): δ 9.44 (br s, 1H), 8.11 (d, *J* = 7.70 Hz, 1H), 7.44 (dd, *J* = 8.07, 12.65 Hz, 2H), 7.14–7.19 (m, 1H), 7.10 (d, *J* = 8.07 Hz, 1H), 7.00 (td, *J* = 7.45, 19.03 Hz, 2H), 6.91 (t, *J* = 7.34 Hz, 1H), 3.83 (s, 3H), 2.26 (br s, 6H), 2.06 (br s, 3H), 1.71–1.85 (m, 6H). ^13^C-NMR (DMSO-*d*_6_): δ 185.2, 168.0, 150.5, 130.1, 127.2, 125.4, 122.1, 121.0, 120.9, 120.8, 119.4, 114.7, 114.3, 111.9, 111.0, 107.6, 56.2, 39.2, 36.9, 36.9, 28.7. ESI-HRMS (+): *m*/*z* [M + H]^+^ calculated for C_27_H_28_N_2_O_3_^+^, 429.2173, found 429.2171; [M + Na]^+^ calculated for C_27_H_28_N_2_O_3_Na^+^, 451.1992, found 451.1990.

*2-(2-Adamantane-3H-indol-3-yl)-2-oxo-N-(3-(trifluoromethyl)phenyl)acetamide* (**5i**): yellow solid, yield 88.0%. ^1^H-NMR (DMSO-*d*_6_): δ 8.22 (s, 1H), 8.02 (d, *J* = 8.07 Hz, 1H), 7.65 (t, *J* = 7.98 Hz, 1H), 7.51 (t, *J* = 7.15 Hz, 2H), 7.35 (d, *J* = 8.07 Hz, 1H), 7.23–7.27 (m, 1H), 7.17 (d, *J* = 7.34 Hz, 1H), 7.09 (t, *J* = 7.52 Hz, 1H), 6.98–7.02 (m, 1H), 2.27 (br s, 6H), 2.09 (br s, 3H), 1.73–1.86 (m, 6H). ^13^C-NMR (DMSO-*d*_6_): δ 183.8, 168.3, 140.0, 137.8, 130.8, 130.1(d, *J* = 31.9 Hz), 129.4, 128.7, 125.8, 123.1(q, *J* = 280.6 Hz), 119.0, 116.1(q, *J* = 3.3 Hz), 113.8, 107.5, 38.8, 36.6, 28.4, 21.5. ESI-HRMS (+): *m*/*z* [M + H]^+^ calculated for C_27_H_25_F_3_N_2_O_2_^+^, 467.1941, found 467.1943; [M + Na]^+^ calculated for C_27_H_25_F_3_N_2_O_2_Na^+^, 489.1760, found 489.1762.

*N-(4-Methoxyphenyl)-2-(2-adamantane-3H-indol-3-yl)-2-oxoacetamide* (**5j**): yellow solid, yield 89.5%. ^1^H-NMR (DMSO-*d*_6_): δ 10.60 (s, 1H), 7.68 (d, *J* = 8.99 Hz, 2H), 7.54 (d, *J* = 7.89 Hz, 1H), 7.41 (d, *J* = 8.25 Hz, 1H), 7.22–7.28 (m, 2H), 7.17 (d, *J* = 7.34 Hz, 2H), 7.10–7.16 (m, 2H), 7.01–7.07 (m, 1H), 6.97 (d, *J* = 8.99 Hz, 2H), 3.76 (s, 3H), 2.30 (s, 3H), 2.26 (br s, 6H), 2.10 (br s, 3H), 1.74–1.87 (m, 6H). ^13^C-NMR (DMSO-*d*_6_): δ 185.6, 167.9,168.2, 158.6, 135.3, 134.7, 128.6,127.1, 126.6, 122.3, 122.1, 119.5, 113.1, 107.9, 55.7,38.7, 36.5, 36.5, 28.4, 19.05. ESI-HRMS (+): *m*/*z* [M + H]^+^ calculated for C_27_H_28_N_2_O_3_^+^, 429.2173, found 429.2168; [M + Na]^+^ calculated for C_27_H_28_N_2_O_3_Na^+^, 451.1992, found 451.1986.

*N-(2,4-Dimethylphenyl)-2-(2-adamantane-1H-indol-3-yl)-2-oxoacetamide* (**5k**): yellow solid, yield 81.5%. ^1^H-NMR (DMSO-*d*_6_): δ 10.08 (br s, 1H), 7.55 (m, 2H), 7.38 (d, *J* = 7.89 Hz ,1H), 7.16 (t, *J* *=* 7.34 Hz, 1H), 7.11 (s, 1H ), 7.09 (d, *J* = 4.77 Hz,1H), 7.07 (s, 1H), 2.30 (s, 3H), 2.37 (s, 6H), 2.23 (s, 3H), 2.11 (s, 3H), 1.83 (m, 6H). ^13^C-NMR (DMSO-*d*_6_): δ 186.2, 167.9, 158.4, 135.5, 135.0, 133.0, 132.9, 131.6, 128.1, 127.2, 125.7, 122.3, 121.9, 119.7, 113.0, 108.0, 38.8, 36.6, 36.5, 28.4, 21.0, 18.1. ESI-HRMS (+): *m*/*z* [M + H]^+^ calculated for C_28_H_30_N_2_O_2_^+^, 427.238, found 427.2384; [M + Na]^+^ calculated for C_28_H_30_N_2_O_2_Na^+^, 449.2199, found 449.2201.

*N-(4-Chloro-2-methylphenyl)-2-(2-adamantane-3H-indol-3-yl)-2-oxoacetamide* (**5l**): yellow solid, yield 87.5%. ^1^H-NMR (DMSO-*d*_6_): δ 7.56 (dd, *J* = 8.25, 13.57 Hz, 2H), 7.49 (d, *J* = 8.07 Hz, 1H), 7.39 (s, 1H), 7.34 (dd, *J* = 2.02, 8.44 Hz, 1H), 7.15 (t, *J* = 7.61 Hz, 1H), 7.08–7.11 (m, 1H), 2.26 (s, 9H), 2.10 (br s, 3H), 1.75–1.87 (m, 6H). ^13^C-NMR (DMSO-*d*_6_): δ 185.6, 167.9, 168.2, 158.6, 135.3, 134.7, 130.6, 128.6, 127.1, 126.6, 122.3, 122.1, 119.5, 113.1, 107.9, 38.7, 36.5, 36.5, 28.4, 19.05, 18.3. ESI-HRMS (+): *m*/*z* [M + H]^+^ calculated for C_27_H_27_ClN_2_O_2_^+^, 447.1834, found 447.1832; [M + Na]^+^ calculated for C_27_H_27_ClN_2_O_2_Na^+^, 469.1653, found 469.1649.

*2-(2-Adamantane-3H-indol-3-yl)-N-(2-methyl-4-nitrophenyl)-2-oxoacetamide* (**5m**): yellow solid, yield 90.5%. ^1^H-NMR (DMSO-*d*_6_): δ 8.20 (brs, 1H), 8.17 (d, *J* = 8.62 Hz, 1H), 8.02 (d, *J* = 7.70 Hz, 1H), 7.56 (d, *J* = 8.07 Hz, 1H), 7.46 (d, *J* = 8.07 Hz, 1H), 7.16 (t, *J* = 7.52 Hz, 1H), 7.06–7.11 (m, 1H), 2.39 (s, 3H), 2.27 (br s, 6H), 2.10 (br s, 3H), 1.75–1.87 (m, 6H); ^13^C-NMR (DMSO-*d*_6_): δ 185.1, 168.2, 158.7, 144.3, 142.9, 134.8, 133.0, 127.9, 126.1, 124.6, 122.5, 122.4, 122.2, 119.4, 113.1, 107.9, 38.7, 36.5, 36.5, 28.4, 18.3. ESI-HRMS (+): *m*/*z* [M + H]^+^ calculated for C_27_H_27_N_3_O_4_^+^, 458.2074, found 458.2072; [M + Na]^+^ calculated for C_27_H_27_N_3_O_4_Na^+^, 480.1894, found 480.1892. methyl benzoate

*Methyl 3-methyl-4-(2-(2-adamantane-3H-indol-3-yl)-2-oxoacetamido)benzoate* (**5n**): yellow solid, yield 89.5%. ^1^H-NMR (DMSO-*d*_6_): δ11.82 (s, 1H), 10.39 (s, 1H), 7.90 (s, 1H), 7.88 (d, *J* = 1.65 Hz, 1H), 7.82–7.87 (m, 1H), 7.56 (d, *J* = 7.89 Hz, 1H), 7.49 (d, *J* = 8.07 Hz, 1H), 7.12–7.16 (m, 1H), 7.08–7.12 (m, 1H), 3.86 (s, 3H), 2.34 (s, 3H), 2.24–2.29 (m, 6H), 2.11 (br s, 3H), 1.75–1.87 (m, 6H); ^13^C-NMR (DMSO-*d*_6_): δ 185.4, 167.8, 166.4, 158.5, 140.4, 134.7, 132.2, 132.0, 129.4, 128.7, 127.9, 126.8, 125.8, 124.7, 122.5, 122.2, 119.5, 113.0, 107.9, 52.5, 38.7, 36.5, 28.4, 21.5, 18.2. ESI-HRMS (+): *m*/*z* [M + H]^+^ calculated for C_29_H_30_N_2_O_4_^+^, 471.2278, found 471.2278; [M + Na]^+^ calculated for C_29_H_30_N_2_O_4_Na^+^, 493.2098, found 493.2096.

*N-(3,4-Dimethoxyphenyl)-2-(2-adamantane-3H-indol-3-yl)-2-oxoacetamide* (**5o**): yellow solid, yield 87.9%. ^1^H-NMR (DMSO-*d*_6_): δ 10.45 (br s, 1H), 7.49 (d, *J* = 8.07 Hz, 1H), 7.40 (d, *J* = 2.38 Hz, 1H), 7.35–7.37 (m, 1H), 7.05 (t, *J* = 7.52 Hz, 1H), 6.94–6.99 (m, 2H), 3.75 (d, *J* = 8.25 Hz, 6H), 2.27(br s, 6H), 2.08 (br s, 3H), 1.72–1.86 (m, 6H). ^13^C-NMR (DMSO-*d*_6_): δ 167.7, 149.2, 145.8, 137.8, 132.8, 129.4, 128.7, 125.8, 121.6, 121.5, 111.9, 113.8, 112.7, 112.2, 107.7, 105.2, 56.2, 55.8, 39.6, 38.9, 36.7, 28.5. ESI-HRMS (+): *m*/*z* [M + H]^+^ calculated for C_28_H_30_N_2_O_4_^+^, 459.2278, found 459.2277; [M + Na]^+^ calculated for C_28_H_30_N_2_O_4_Na^+^, 481.2098, found 481.2098.

*N-(3,5-Dimethoxyphenyl)-2-(2-adamantane-3H-indol-3-yl)-2-oxoacetamide* (**5p**): yellow solid, yield 89.5%. ^1^H-NMR (DMSO-*d*_6_): δ 10.68 (s, 1H), 7.55 (d, *J* = 8.07 Hz, 1H), 7.39 (d, *J* = 8.07 Hz, 1H), 7.12–7.16 (m, 1H), 7.04-7.08 (m, 1H), 7.01 (s, 1H), 7.01 (s, 1H), 6.33 (t, *J* = 2.20 Hz, 1H), 3.74 (s, 6H), 2.26 (br s, 6H), 2.10 (br s, 3H), 1.74–1.86 (m, 6H). ^13^C-NMR (DMSO-*d*_6_): δ 185.3, 167.6, 161.1, 158.6, 140.6, 134.8, 129.4, 127.9, 122.4, 122.3, 119.4, 113.0, 107.8, 98.7, 96.2, 55.6, 38.7, 36.5, 36.5, 28.4. ESI-HRMS (+): *m*/*z* [M + H]^+^ calculated for C_28_H_30_N_2_O_4_^+^, 459.2278, found 459.2276; [M + Na]^+^ calculated for C_28_H_30_N_2_O_4_Na^+^, 481.2098, found 481.2096.

*N-(4-Chloro-3-methoxyphenyl)-2-(2-adamantane-3H-indol-3-yl)-2-oxoacetamide* (**5q**): yellow solid, yield 85.7%. ^1^H-NMR (DMSO-*d*_6_): δ 7.59 (s, 1H), 7.54 (d, *J* = 7.89 Hz, 1H), 7.43 (s, 2H), 7.38 (d, *J* = 8.07 Hz, 1H), 7.15–7.19 (m, 1H), 7.13 (t, *J* = 7.70 Hz, 1H), 7.02–7.07 (m, 1H), 3.79–3.88 (m, 3H), 2.26 (br s, 6H), 2.10 (br s, 3H), 1.74–1.86 (m, 6H). ^13^C-NMR (DMSO-*d*_6_): δ 184.8, 167.7, 155.1, 139.2, 137.8, 130.5, 129.0, 125.8, 122.2, 119.3, 116.3, 113.3, 112.9, 107.7, 104.8, 56.3, 40.1, 38.8, 36.6, 28.4, 21.5. ESI-HRMS (+): *m*/*z* [M + H]^+^ calculated for C_27_H_27_ClN_2_O_3_^+^, 463.1783, found 463.1781; [M + Na]^+^ calculated for C_27_H_27_ClN_2_O_3_Na^+^, 485.1602, found 485.1601.

*N-(3-Chloro-4-fluorophenyl)-2-(2-adamantane-3H-indol-3-yl)-2-oxoacetamide* (**5r**): yellow solid, yield 85.9%. ^1^H-NMR (DMSO-*d*_6_): δ 11.85 (br s, 1H), 11.01 (br s, 1H), 8.07 (dd, *J* = 2.57, 6.79 Hz, 1H), 7.68–7.72 (m, 1H), 7.56 (d, *J* = 8.07 Hz, 1H), 7.47 (t, *J* = 9.08 Hz, 1H), 7.36 (d, *J* = 8.07 Hz, 1H), 7.13–7.16 (m, 1H), 7.05–7.10 (m, 1H), 2.26 (br s, 6H), 2.10 (br s, 3H), 1.75–1.85 (m, 6H). ^13^C-NMR (DMSO-*d*_6_): *δ* 184.9, 167.4, 158.8 (d, *J* = 244.3 Hz), 154.1, 136.2, 134.7, 129.4, 128.7, 125.8, 122.5 (d, *J* = 15.4 Hz), 121.5, 120.6 (d, *J* = 6.6 Hz), 119.3, 117.8, 113.1, 107.7, 38.7, 36.5, 28.3, 21.5. ESI-HRMS (+): *m*/*z* [M + H]^+^ calculated for C_26_H_24_ClFN_2_O_2_^+^, 451.1583, found 451.1584; [M + Na]^+^ calculated for C_26_H_24_ClFN_2_O_2_Na^+^, 473.1403, found 473.1405.

*N-(4-Bromo-2-fluorophenyl)-2-(2-adamantane-3H-indol-3-yl)-2-oxoacetamide* (**5s**): yellow solid, yield 88.5%. ^1^H-NMR (DMSO-*d*_6_): δ 7.88 (br s, 1H), 7.68 (d, *J* = 9.90 Hz, 1H), 7.55 (d, *J* = 8.07 Hz, 1H), 7.43–7.52 (m, 2H), 7.14 (t, *J* = 7.15 Hz, 1H), 7.04–7.09 (m, 1H), 2.26 (br s, 6H), 2.10 (br s, 3H), 1.71–1.87 (m, 6H). ^13^C-NMR (DMSO-*d*_6_) : δ 184.6, 168.0, 155.0 (d, *J* = 249.8 Hz), 129.4, 128.7, 128.2, 128.2, 128.1, 126.9, 125.8, 122.1(d, *J* = 29.7 Hz), 119.8(d, *J* = 23.1Hz), 119.3, 113.2, 107.8, 38.7, 36.6, 36.5, 28.4. ESI-HRMS (+): *m*/*z* [M + H]^+^ calculated for C_26_H_24_BrFN_2_O_2_^+^, 495.1078, found 495.1079; [M + Na]^+^ calculated for C_26_H_24_BrFN_2_O_2_Na^+^, 517.0897, found 517.0896.

*N-(5-Bromo-2-fluorophenyl)-2-(2-adamantane-3H-indol-3-yl)-2-oxoacetamide* (**5t**): yellow solid, yield 85.6%. ^1^H-NMR (DMSO-*d*_6_): δ 8.17 (br s, 1H), 7.53 (d, *J* = 8.07 Hz, 1H), 7.46 (d, *J* = 8.07 Hz, 1H), 7.38 (br s, 1H), 7.30 (t, *J* = 9.44 Hz, 1H), 7.12 (t, *J* = 7.24 Hz, 1H), 7.03–7.08 (m, 1H), 2.25 (br s, 6H), 2.08 (br s, 3H), 1.74-1.85 (m, 6H). ^13^C-NMR (DMSO-*d*_6_): δ 170.3, 168.6, 154.1(d, *J* = 247.6 Hz), 137.8, 129.4, 128.7, 128.2, 127.3, 125.8, 122.0(d, *J* = 28.6 Hz), 119.4, 118.3(d, *J* = 21.0 Hz), 116.2, 113.2, 107.9, 38.7, 36.6, 36.5, 28.4. ESI-HRMS (+): *m*/*z* [M + H]^+^ calculated for C_26_H_24_BrFN_2_O_2_^+^, 495.1078, found 495.1079; [M + Na]^+^ calculated for C_26_H_24_BrFN_2_O_2_Na^+^, 517.0897, found 517.0893.

*N-(2-Fluoro-4-iodophenyl)-2-(2-adamantane-3H-indol-3-yl)-2-oxoacetamide* (**5u**): yellow solid, yield 86.5%. ^1^H-NMR (DMSO-*d*_6_): δ 7.73 (d, *J* = 9.72 Hz, 2H), 7.61 (d, *J* = 7.89 Hz, 1H), 7.51 (d, *J* = 7.89 Hz, 1H), 7.45 (d, *J* = 8.07 Hz, 1H), 7.06–7.12 (m, 1H), 7.02 (t, *J* = 7.43 Hz, 1H), 2.26 (br s, 6H), 2.08 (br s, 3H), 1.74–1.86 (m, 6H). ^13^C-NMR (DMSO-*d*_6_): δ 170.3, 168.6, 154.9 (d, *J* = 253.1 Hz), 137.8, 134.0, 129.4, 128.7, 127.2, 125.8, 125.0 (d, *J* = 22.0 Hz), 121.7 (d, *J* = 22.1 Hz), 119.3, 113.6, 107.8, 38.8, 36.7, 36.6, 28.5. ESI-HRMS (+): *m*/*z* [M + H]^+^ calculated for C_26_H_24_FIN_2_O_2_^+^, 543.0939, found 543.0939; [M + Na]^+^ calculated for C_26_H_24_FIN_2_O_2_Na^+^, 565.0759, found 565.0757.

*2-(2-Adamantane-3H-indol-3-yl)-2-oxo-N-(pyridin-3-yl)acetamide* (**5v**): yellow solid, yield 88.6%. ^1^H-NMR (DMSO-*d*_6_): δ 11.93 (br s, 1H), 11.05 (s, 1H), 8.93 (d, *J* = 2.02 Hz, 1H), 8.38 (d, *J* = 4.03 Hz, 1H), 8.21 (d, *J* = 8.25 Hz, 1H), 7.59 (d, *J* = 7.89 Hz, 1H), 7.45 (dd, *J* = 4.77, 8.07 Hz, 1H), 7.39 (d, *J* = 8.07 Hz, 1H), 7.15 (t, *J* = 7.61 Hz, 1H), 7.04–7.09 (m, 1H), 2.27 (br s, 6H), 2.10 (br s, 3H), 1.74–1.86 (m, 6H). ^13^C-NMR (DMSO-*d*_6_): δ 185.0, 167.8, 158.8, 145.5, 141.8, 135.7, 134.8, 127.8, 127.3, 124.4, 122.5, 122.4, 119.3, 113.2, 107.7, 38.7, 36.5, 36.5, 28.3. ESI-HRMS (+): *m*/*z* [M + H]^+^ calculated for C_25_H_25_N_3_O_2_^+^, 400.202, found 400.2017; [M + Na]^+^ calculated for C_25_H_25_N_3_O_2_Na^+^, 422.1839, found 422.1836.

*2-(2-Adamantane-3H-indol-3-yl)-2-oxo-N-(pyridin-2-yl)acetamide* (**5w**): yellow solid, yield 90.6%. ^1^H-NMR (DMSO-*d*_6_): δ 11.93 (brs, 1H), 11.04 (s, 1H), 8.92 (d, *J* = 2.20 Hz, 1H), 8.38 (d, J = 4.59 Hz, 1H), 8.21 (d, *J* = 8.25 Hz, 1H), 7.59 (d, *J* = 8.07 Hz, 1H), 7.45 (dd, *J* = 4.68, 8.16 Hz, 1H), 7.39 (d, *J* = 8.25 Hz, 1H), 7.15 (t, *J* = 7.61 Hz, 1H), 7.04–7.09 (m, 1H), 2.27 (brs, 6H), 2.10 (brs, 3H), 1.74–1.87 (m, 6H). ^13^C-NMR (DMSO-*d*_6_): δ 185.0, 167.8, 158.8, 145.5, 141.8, 135.7, 134.8, 127.8, 127.3, 124.4, 122.5, 122.4, 119.3, 113.2, 107.7, 38.7, 36.5, 36.5, 28.3. ESI-HRMS (+): *m*/*z* [M + H]^+^ calculated for C_25_H_25_N_3_O_2_^+^, 400.202, found 400.2016; [M + Na]^+^ calculated for C_25_H_25_N_3_O_2_Na^+^, 422.1839, found 422.1835.

*2-(2-Adamantane-3H-indol-3-yl)-2-oxo-N-(pyrimidin-5-yl)acetamide* (**5x**): yellow solid, yield 90.1%. ^1^H-NMR (DMSO-*d*_6_): δ 9.16 (s, 2H), 8.97 (s, 1H), 7.54–7.59 (m, 1H), 7.38 (d, *J* = 8.25 Hz, 1H), 7.11–7.15 (m, 1H), 7.03–7.08 (m, 1H), 2.26 (br s, 6H), 2.09 (br s, 3H), 1.75–1.85 (m, 6H). ^13^C-NMR (DMSO-*d*_6_): δ 184.5, 168.2, 153.9, 148.4, 147.67, 129.3, 128.7, 128.1, 125.8, 122.3, 119.3, 113.4,107.6, 38.7, 36.5, 36.5, 28.4, 19.1. ESI-HRMS (+): *m*/*z* [M + H]^+^ calculated for C_24_H_24_N_4_O_2_^+^, 401.1972, found 401.1969; [M + Na]^+^ calculated for C_24_H_24_N_4_O_2_Na^+^, 423.1791, found 423.1785.

*N-(5-tert-Butylisoxazol-3-yl)-2-(2-adamantane-3H-indol-3-yl)-2-oxoacetamide* (**5y**): yellow solid, yield. 86.8%. ^1^H-NMR (DMSO-*d*_6_): δ 11.79 (br s, 2H), 7.56 (d, *J* = 7.89 Hz, 1H), 7.38 (d, *J* = 8.07 Hz, 1H), 7.16 (t, *J* = 7.52 Hz, 1H), 7.08–7.12 (m, 1H), 6.72 (s, 1H), 2.24 (br s, 6H), 2.10 (br s, 3H), 1.75–1.86 (m, 6H), 1.35 (s, 9H). ^13^C-NMR (DMSO-*d*_6_): δ 184.2, 181.5, 167.7, 158.9, 157.8, 134.8, 127.7, 122.7, 122.5, 122.4, 119.0, 113.2, 107.5, 99.8, 38.5, 36.5, 33.1, 28.9, 28.3. ESI-HRMS (+): *m*/*z* [M + H]^+^ calculated for C_27_H_31_N_3_O_3_^+^, 446.2438, found 446.2433; [M + Na]^+^ calculated for C_27_H_31_N_3_O_3_Na^+^, 468.2258, found 468.2256.

### 3.5. Cell Culture

All of the human cancer cell lines were obtained from the American Type Culture Collection (ATCC, Manassas, VA, USA) and grown in DMEM culture medium containing 10% fetal bovine serum (*v*/*v*) in 5% CO_2_ at 37 °C.

### 3.6. Cytotoxicity against Cancer Cell Lines

Confluent cancer cells in good state were cultured in 96-well plates (5–6 ×10^3^ cells/well) and treated with various concentrations of compounds at 37 °C for 24 h. Then, the cells were incubated with 20μL of 5 mg/mL 3-(4,5-dimethyl-2-thiazolyl)-2,5-diphenyl-2*H*-tetrazolium bromide, MTT, (Sigma-Aldrich, Saint Louis, MO, USA) reagent at 37 °C for 4 h. The supernatant was removed, and cells were dissolved in 150 μL dimethyl sulfoxide and shaken for 5 min. Finally, the light absorption (OD) of the dissolved cells was measured at 490 nm.

### 3.7. Western Blot Analysis

Equal amounts of the lysates were electrophoresed on 8% SDS-PAGE gel and transferred onto PVDF membranes (Roche, Shanghai, China). After blocked with 5% nonfat milk in TBST (20 mM Tris-HCl (pH 7.4), 150 mM NaCl and 0.1% Tween 20) for 1 h, The membranes were incubated with various primary antibodies overnight and secondary antibodies for 2 h, finally detected using ECL system. Proteins were detected with the following antibodies: Rabit anti-Parp mAb (46D11, Cell Signaling Technology, Shanghai, China), Mouse anti-β-actin mAb (sc-8432, Santa Cruz, Shanghai, China).

### 3.8. A Flow Cytometry Assay

Hela cells were cultured in 6-well plate and treated with various compounds in serum free medium for 24 h. Then, the cells were detached by trypsin and fixed in 70% cold ethanol overnight at 4 °C. The next day, the cells were centrifuged in 3000 rpm for 53 min, washed twice in PBS, and incubated with DNase A (100 µg/mL) and propidium iodide (PI) solution (50 µg/mL) at room temperature for 30 min. The cell cycle was detected by flow cytometry (Beckman Coulter, Pasadena, CA, USA). 

### 3.9. Colony Formation Assay

Hela cells were cultured in 6-well plate (200 cells/well) and treated with various compounds in 1% serum medium for 6 days. Then fixed with 4% paraformaldehyde and stained with 0.1% crystal violet.

### 3.10. Caspase Activity Assay

The activities of caspase-3, caspase-8 and caspase-9 were measured using the caspase activity kit (Beyotime Biotechnology, Shanghai, China) according to the manufacturer’s instructions. Briefly, testis lysates were prepared after treatment. 50 μL testis lysate, 50 μL reaction buffer and 5 μL caspases substrate were added, incubated at 37 °C for 3 h. Samples were measured with an ELISA Reader (Bio-Rad instrument Group, Hercules, CA, USA) at an absorbance of 405 nm. All the experiments were carried out in triplicates.

## 4. Conclusions 

In this paper, we synthesized and conducted a biological evaluation of a new series of *N*-substituted 2-(2-(adamantan-1-yl)-1*H*-indol-3-yl)-2-oxoacetamide derivatives as potential anticancer agents. The synthetic method was relatively simple, and the compounds were produced in high yields and easily purified. Compound **5r** showed more significant inhibitory activity against HepG2 cells than other compounds, with an IC_50_ value of 10.56 ± 1.14 μΜ, as well as excellent selectivity toward HepG2 over HeLa and MCF-7 cells. Western blot analysis and flow cytometry assay demonstrated that compound **5r** could arrest the cell cycle, activate caspase-8 and caspase-3 and induce cell apoptosis. However, determining its roles in preventing cancer still require further intensive study.

## Figures and Tables

**Table 1:** Inhibition of humor cancer cell lines (Hela, MCF7 and HepG2) by *N*-substituted 2-(2-(adamantan-1-yl)-1*H*-indol-3-yl)-2-oxoacetamide derivatives.

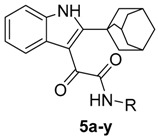

Compound	Structure	MTT (IC_50_, μM)		
	R	Hela	MCF7	HepG2
**5a**	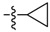	>100	>100	>100
**5b**	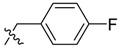	62.13 ± 3.45	>100	56.46 ± 2.71
**5c**	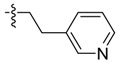	43.21 ± 1.67	46.12 ± 2.15	37.87 ± 0.87
**5d**	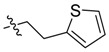	42.14 ± 2.61	46.19 ± 1.34	35.67 ± 1.69
**5e**	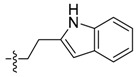	>100	>100	61.12 ± 2.54
**5f**	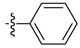	17.65 ± 1.54	24.24 ± 1.09	20.89 ± 0.87
**5g**	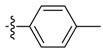	42.12 ± 1.94	40.67 ± 1.48	35.65 ± 1.02
**5h**	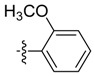	41.21 ± 1.54	38.91 ± 1.04	25.69 ± 2.01
**5i**	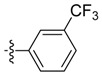	28.23 ± 1.16	26.11 ± 0.98	25.14 ± 0.61
**5j**	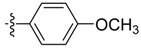	>100	29.25 ± 1.90	21.34 ± 0.98
**5k**	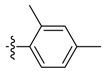	45.61 ± 1.67	36.15 ± 1.23	36.75 ± 1.85
**5l**	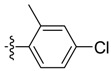	65.43 ± 1.15	68.21 ± 2.78	43.21 ± 1.45
**5m**	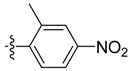	85.34 ± 1.54	76.34 ± 1.25	57.17 ± 2.15
**5n**	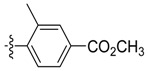	54.32 ± 1.15	41.24 ± 1.12	40.13 ± 2.54
**5o**	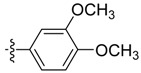	35.13 ± 1.98	45.13 ± 2.32	43.12 ± 2.32
**5p**	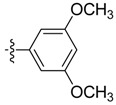	40.14 ± 1.56	37.45 ± 0.67	22.24 ± 1.68
**5q**	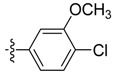	36.56 ± 1.12	35.65 ± 1.41	38.65 ± 1.63
**5r**	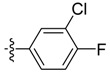	16.12 ± 1.54	12.54 ± 1.15	10.56 ± 1.14
**5s**	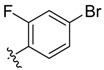	34.35 ± 1.45	45.83 ± 2.41	35.67 ± 1.14
**5t**	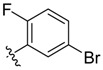	37.32 ± 1.78	49.32 ± 2.16	36.76 ± 0.79
**5u**	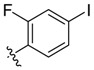	47.32 ± 1.78	56.14 ± 2.38	42.15 ± 2.53
**5v**	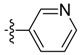	78.34 ± 0.56	56.34 ± 1.56	41.22 ± 2.19
**5w**	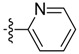	32.15 ± 2.21	43.12 ± 2.10	46.14 ± 1.45
**5x**	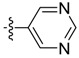	42.15 ± 2.23	53.12 ± 2.16	41.14 ± 1.92
**5y**	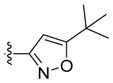	31.47 ± 1.24	29.25 ± 1.90	21.34 ± 0.98

## Supplementary Materials

Supplementary materials can be accessed at: http://www.mdpi.com/1420-3049/21/5/530/s1.
