# Supplementary material for: Novel N-Substituted 2-(2-(Adamantan-1-yl)-1H-Indol-3-yl)-2-Oxoacetamide Derivatives: Synthesis and Biological Evaluation
Source: Molecules. 2016 May 5;21(5):530. doi: 10.3390/molecules21050530 (PMC6273615; doi:10.3390/molecules21050530)
Supplement: Supplementary file 1 [file molecules-21-00530-s001.pdf]

Supplementary Materials: Novel *N*-Substituted 2-(2-(Adamantan-1-yl)-1*H*-Indol-3-yl)-2-Oxoacetamide Derivatives: Synthesis and Biological Evaluation

Hongyu Hu, Xudong Yu, Fei Wang, Chunrong Lin, Jinzhang Zeng, Yingkun Qiu, Meijuan Fang and Zhen Wu

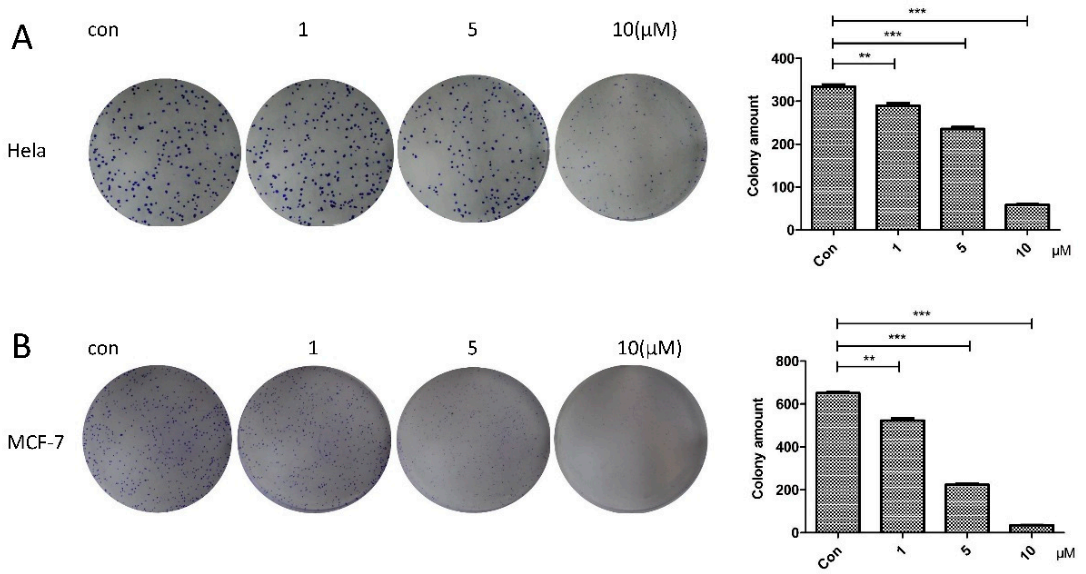

Figure S1. Effects of compound **5r** on colony-formation in Hela (**A**) and MCF-7 (**B**) cells. Compound **5r** inhibits the colony-forming activity of Hela and MCF-7 cells. Cells were grown in 6-well plates for 6 days and treated with compound **5r** (1, 5 and 10 μM). Colonies of Hela and MCF-7 cells decreased after compound 5r treatment in a dose dependent manner (** $p < 0.01$ and *** $p < 0.001$ compared with the control, *t*-test).

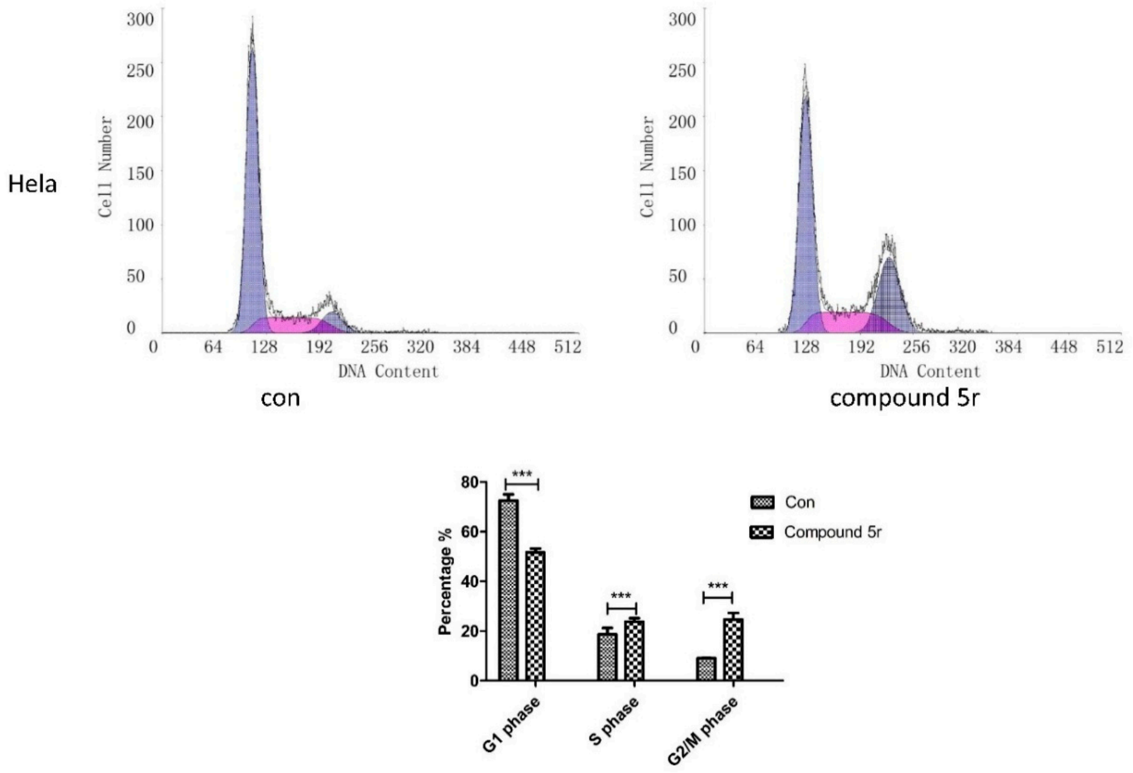

Figure S2. Effects of compound **5r** on cell-cycle distribution in Hela cells. A flow cytometry assay was performed to examine cell cycle arrest. Hela cells were treated with 10 μM compound **5r** for 12 h. Compound **5r** induced a significant accumulation of cell-cycle arrest (*** $p < 0.001$ compared with the control, *t*-test).

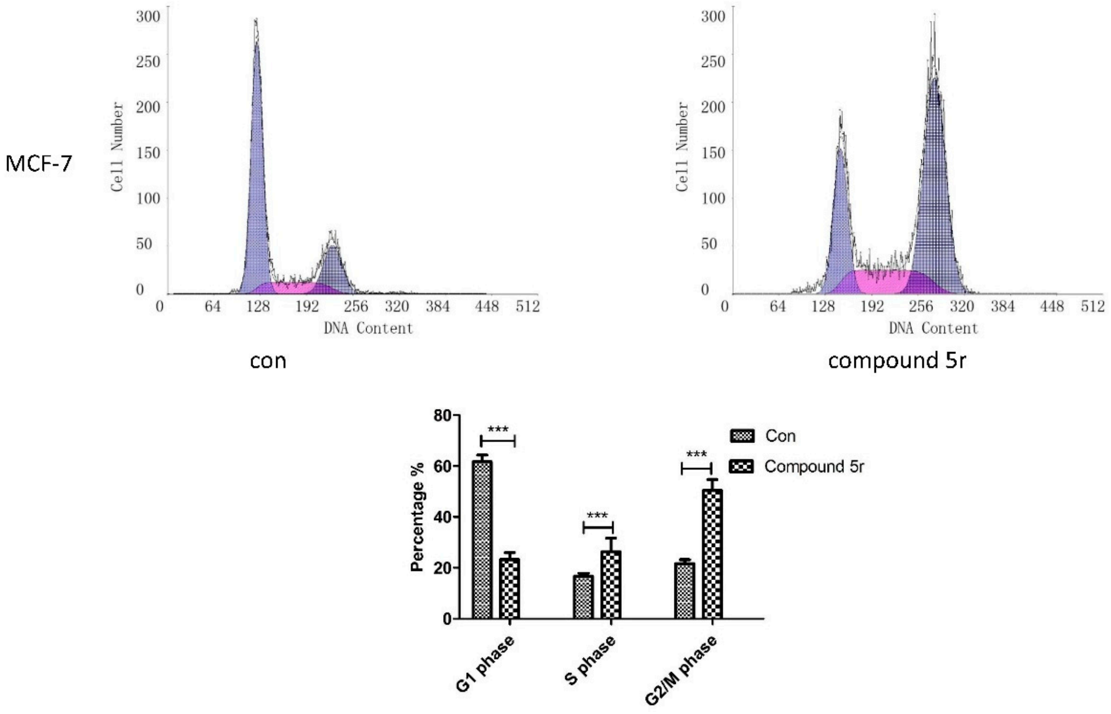

Figure S3. Effects of compound **5r** on cell-cycle distribution in MCF-7 cells. A flow cytometry assay was performed to examine cell cycle arrest MCF-7 cells were treated with 10 μM compound **5r** for 12 h. Compound **5r** induced a significant accumulation of cell-cycle arrest (*** $p < 0.001$ compared with the control, *t*-test).
